# Effect of the COVID-19 Vaccine on the Menstrual Cycle among Females in Saudi Arabia

**DOI:** 10.4314/ejhs.v32i6.4

**Published:** 2022-11

**Authors:** Fadi S I Qashqari, Maryam Dahlawi, Hamza M Assaggaf, Radi Alsafi, Abdulrahim Gari, Abdulrahman Abudawood, Amal Al-Doboke, Seham Alsulami, Rahaf Bukhari, Shaza Adnan Majeed, Elaf Abdullah Salih, Mohammad Alfelali, Hatim Makhdoom, Naif A Jalal

**Affiliations:** 1 Department of Microbiology, College of Medicine, Umm Al-Qura University, Makkah 24381, Saudi Arabia; 2 College of Medicine, Umm Al-Qura University, Makkah 24381, Saudi Arabia; 3 Department of Laboratory Medicine, Faculty of Applied Medical Sciences, Umm Al-Qura University, Makkah 21955, Saudi Arabia; 4 Department of Obstetrics and Gynecology, Faculty of Medicine, Umm Al-Qura University, Makkah, Saudi Arabia; 5 Department of Obstetrics and Gynecology, King Faisal Specialist Hospital and Research Center, Jeddah, Saudi Arabia; 6 Department of Family and Community Medicine, Rabigh Medical College, King Abdulaziz University, Jeddah, Saudi Arabia; 7 Makkah healthcare cluster- Maternity and children hospital; 8 Department of Obstetrics and Gynecology, International Medical Center, Jeddah, Saudi Arabi; 9 Department of Family and Community Medicine, Faculty of Medicine in Rabigh, King Abdulaziz University, Jeddah, Saudi Arabia; 10 Applied Medical Sciences College, Laboratory Technology Department, Taibah University, Almadinah Almunwarah, Jeddah, Saudi Arabia

**Keywords:** COVID-19 Vaccine, Menstrual Cycle, Menstrual irregularities, Females, Women, Saudi Arabia

## Abstract

**Background:**

The number of reports of menstrual changes after COVID-19 vaccination in the Saudi population is still unknown. Therefore, this study aimed to assess the effect of the COVID-19 vaccine(Pfizer, AstraZeneca, and Moderna) on the menstrual cycle among females in Saudi Arabia.

**Methods:**

This descriptive cross-sectional study was conducted in Saudi Arabia at Umm Al-Qura University (UQU) from August 2021 to February 2022. Data was collected through a previously validated online questionnaire.

**Results:**

A total of 2338 participants who received the first dose of the COVID-19 vaccine participated in this study; 1606 (68.7%) of them received the second dose in addition to the first. The mean age of the study participants was 35.4±9.5 years. No significant associations were found between the type of COVID-19 vaccine and the impact on the menstrual cycle, either for the first or second dose (P-values > 0.05). A significant association was found only between the first dose vaccination day and the impact on the menstrual cycle in the second question of “After receiving the COVID-19 vaccine, your next period was” (P-value ≤ 0.05). Significant associations were found between the second dose vaccination day and the impact on the menstrual cycle in the first and second questions of “After receiving the COVID-19 vaccine, your next period was”, and “After receiving the first dose, your next period was,” respectively (P-values ≤ 0.05).

**Conclusion:**

The study found a potential association between the COVID-19 vaccine and menstrual cycle irregularities, which could impact females' quality of life.

## Introduction

There are multiple plausible biological mechanisms to explain a relationship between a critical immune challenge like a vaccine and the menstrual cycle (1); the menstrual cycle can be affected by immune activation in response to various stimuli, including immunological influences on the hormones driving the menstrual cycle or effects mediated by immune cells in the lining of the uterus, which is included in the build-up and breakdown of this tissue during each process (2–4). This immune activation is essential, although it may also produce a cascade of other localized (e.g., soreness at the injection site) or systemic (e.g., fatigue, fever) inflammatory responses.

Studies evaluating vaccines' direct effects on the menstrual cycle are few and far between (1). The first published study on vaccine effects on menstrual cycles dates back to 1913 in New York (5) and concluded a unique relationship between receiving the prophylactic typhoid vaccine and menstrual disturbances in one hundred cases. After ruling out all other apparent causes, 53% showed some type of disturbance, including increased or decreased frequency, volume, and dysmenorrhoea. These disturbances disappeared after six months of receiving the vaccine, suggesting that any such vaccine side-effect was temporary (5). A report of menstrual disorders following immunization with the hepatitis vaccine has also been reported. In a Japanese study conducted in 1982 among 16 hospital employees, 7 reported various menstrual abnormalities, such as decreased volume of menstrual and infrequent or too frequent menstrual(5). A study conducted in 2018 among 29,846 females in Nagoya City, Japan, suggests a possible link between the human papillomavirus (HPV)vaccine and menstrual irregularities (5,6). However, the evidence is noncausal, and relationships might depend on the type of vaccine.

Concerning COVID-19 vaccination, as early as 2021, more than 30 000 reports of menstrual irregularities after COVID-19 vaccination had been reported to the United Kingdom's (U.K.) Medicines and Healthcare Products Regulatory Agency (MHRA) (5,7,8). Moreover, many people in social media reports suggest menstrual disturbances are much more common (9). Although reported changes to the menstrual cycle after vaccination are short-lived and temporary, concerns about a possible association between COVID-19 vaccination and abnormal menstrual cycles may lead to vaccine hesitancy. Research into this potential adverse reaction remains critical to the overall success of the vaccination program (10).

The lack of population-level, prospective evidence about the relationship between COVID-19 vaccination and menstrual cycles limits our ability to sufficiently address these concerns and to counsel individuals who menstruate about what to expect with vaccination (9).

A cohort study in the United States (U.S.)assessing whether COVID-19 vaccination is associated with changes in the cycle or menstrual length in those receiving vaccination as compared with the unvaccinated residents aged 18–45 years found that COVID-19 immunization is associated with a slight change in menstrual cycle length but not menstrual length (9). Another study in the U.K. evaluated the incidence of reports of menstrual changes following COVID-19 vaccination. Following vaccination for COVID-19, they found a menstrual disturbance in 20% of 61 individuals in a U.K. sample (5). Moreover, a study aimed to assess the relationship between vaccination and the occurrence of such disturbances among women aged 18–30 in Norway found a significant increase in menstrual disorders after COVID-19 immunization, particularly for heavier bleeding than usual, longer duration, and short intervals between menstruations (7).

Unfortunately, the number of reports of menstrual changes after COVID-10 vaccination in the Saudi population is still unknown. This stresses the need to examine menstruation irregularities after receiving the COVID-19 vaccination. Therefore, this study aimed to assess the effect of the COVID-19 vaccine on the menstrual cycle among females in Saudi Arabia.

## Methods

**Study design and setting**: This descriptive cross-sectional study was conducted in Saudi Arabia at Umm Al-Qura University (UQU) from August 2021 to February 2022.

**Study population**: All females who are residents in Saudi Arabia, aged 12 and above, and receiving the COVID-19 vaccine were eligible to participate. In contrast, any female who lives outside Saudi Arabia, aged less than 12, or is menopausal was excluded from the study.

**Data collection tool**: Data was collected through a previously validated online questionnaire(10); the questionnaire covered the following four sections: A) The participants' sociodemographic data, including age, residence region, age at which the first menstrual cycle started, if there is any usage of hormones for contraception or any other reason if the female has been diagnosed with any of the following diseases (heavy menstrual bleeding, abnormal menstrual bleeding, menorrhagia, endometriosis, uterine fibrosis, polycystic ovary syndrome, a bleeding or clotting disorder, an autoimmune disease, allergies or asthma). B) Data about the first dose of vaccine and the following associated changes in the menstrual cycle, including the type of the received vaccine, which day of the menstrual cycle was the first dose taken, and the associated changes. C) Same as section B, but it was for the female who received the second dose of the vaccine. D) Questions to assess if there is anything else she has experienced following the COVID-19 vaccine and if menstrual cycle changes have been noticed after any other vaccine.

**Sampling process**: The participants were selected via a random sampling technique from all five regions of Saudi Arabia (Eastern, Western, Northern, Southern, and Central). For data collection, an online survey was distributed using the Google Platform through social media. While 5174 participants began the online survey, 2836 responses were excluded from the data set. A final sample of 2338 individuals was selected as study participants.

**Data analysis:** Data were analyzed using the Statistical Package for Social Science (IBM SPSS) version 24. We used the chi-square test for categorical data analysis. A *P-value*< 0.05 was considered statistically significant.

**Ethical considerations**: Approval was provided by the institutional review board (IRB) of Umm AlQura University (UQU), license no. (HAPO-02-k-012-2021-09-747). Consents were obtained electronically from all participants after the study aims were explained.

## Results

A total of 2338 participants who received the first dose of the COVID-19 vaccine were included in this study; 1606 (68.7%) of them received the second dose in addition to the first. The mean age of the study participants was 35.4±9.5 years. The majority of participants were from theWestern Region, 1111 (47.5%); 579 (24.8%) in the Central region; 380 (16.3%) in the Southern region; 193 (8.3%) in the Eastern region; and 75 (3.2%) in the Northern region. Regarding the menstrual cycle length, 261 (11.2%) participants had their menstrual cycle for less than five days, 1826 (78.1%) for five to seven days, and 251 (10.7%) for more than seven days. 213 (9.1%) had allergies or asthma, 39 (1.7%) had menorrhagia, 36 (1.5%) had uterine fibroids, 27 (1.2%) had heavy menstrual bleeding, 18 (0.8%) had an autoimmune disease, 11 (0.5%) had endometriosis, 10 (0.4%) had abnormal uterine bleeding, and 7 (0.3%) had bleeding or clotting disorders (Table 1).

**Table 1:** Demographic characteristics of the study participants (N=2338)

Variable	N	%
**Age (Mean±std)**	**35.4±9.5**	
**Residence**		
Southern Region	380	16.3
Eastern Region	193	8.3
Northern Region	75	3.2
Western Region	1111	47.5
Central Region	579	24.8
**How prolonged is your normal menstruation (in** **days)**		
5–7 days	1826	78.1
Less than 5 days	261	11.2
More than 7 days	251	10.7
**Have you ever been diagnosed with any of the** **following**		
Heavy menstrual bleeding	27	1.2
Abnormal menstrual bleeding	10	0.4
Menorrhagia	39	1.7
Endometriosis	11	0.5
Uterine fibrosis	36	1.5
A bleeding or clotting disorder	7	0.3
An autoimmune disease	18	0.8
Allergies or Asthma	213	9.1

Table 2 demonstrates the effect of the first dose of the COVID-19 vaccine on the menstrual cycle. Two thousand three hundred thirty-eight participants received the first dose of the COVID-19 vaccine, of which 1694 (72.5%) received Pfizer, 627 (26.8%) acquired AstraZeneca, and 17 (0.7%) received Moderna. 497 (21.3%) of participants received the second dose within 1–7 days of the menstrual cycle; 482 (20.6%) within 8–14 days; 656 (28.1%) within 15–21 days; and 703 (30.1%) after 21 days. The changes in the next period after receiving the first dose were that 1426 (61%) were the same, 547 (24.6%) lighter, and 338 (14.5%) heavier than usual. The next period was on time in 1297 (55.5%), later in 648 (27.7%), and earlier than usual in 393 (16.8%) after receiving the first dose. Menstrual cramps were the same in 1537 (65.7%), worse in 619 (26.5%), and less than usual in 182 (7.8%) following the second dose. The premenstrual syndrome was the same in 1646 (70.4%), worse in 553 (23.7%), and less than usual in 139 (5.9%).

**Table 2:** First dose of COVID-19 vaccine and it is the effect on the menstrual cycle (N = 2338)

Variable	N	%
**Which vaccine did you get for your first dose**		
AstraZeneca	627	26.8
Pfizer	1694	72.5
Moderna	17	0.7
**On which day of your menstrual cycle did you get your first dose (where day 1 is the first day of your** **period)**		
1–7	497	21.3
8–14	482	20.6
15–21	656	28.1
After 21	703	30.1
**Q1. After receiving the first dose, your next period was**		
Lighter than usual	574	24.6
Heavier than usual	338	14.5
The same as usual	1426	61.0
**Q2. After receiving the first dose, your next period was**		
Earlier than usual	393	16.8
On-time	1297	55.5
Later than usual	648	27.7
**Q3. After receiving the first dose, the cramps you experienced in your next period was**		
Worse than usual	619	26.5
Less bad than usual	182	7.8
The same as usual	1537	65.7
**Q4. After receiving the first dose, premenstrual syndrome) PMS you experienced in your next period** **was**		
Worse than usual	553	23.7
Less bad than usual	139	5.9
The same as usual	1646	70.4

Table 3 shows the effect of the second dose of the COVID-19 vaccine on the menstrual cycle. One thousand six hundred six of the participants received the second dose; 1558 (97.0%) of them had a period following the dose; 48 (3.0%) of them had not had a period. Pfizer received 1326 (82.6%) participants, AstraZeneca received 250 (15.6%) participants, and Moderna received 30 (1.9%) participants. 340 (21.2%) of participants received the second dose within 1–7 days of the menstrual cycle, 328 (20.4%) of participants within 8–14 days, 409 (25.5%) of participants within 15–21 days, and 529 (32.9%) of participants after 21 days. The changes in the next period after receiving the second dose were: 997 (62.1 %) as usual, 334 (20.8%) lighter, and 275 (17.1%) heavier than usual. The next period was on time in 923 (57.5%), later in 334 (24.7%), and earlier than usual in 275 (17.9%) after receiving the second dose. Menstrual cramps were the same in 1033 (64.3%), worse in 430 (26.8%), and less than usual in 143 (8.9%) following the second dose. The premenstrual syndrome was the same in 1110(69.1%), worse in 386 (24.0%), and less than usual in 110 (6.8%) following the second dose. The changes following any type of vaccine were not noted in 1565 (97.4%), after another vaccine in 30 (1.9%), after flu vaccine in 9 (0.6%), and after human papillomavirus vaccine in 2 (0.1%).

**Table 3:** Second dose of COVID-19 vaccine and its effect on the menstrual cycle (N = 1606)

Variable	N	%
**Have you had your second dose**		
Yes, and I have had a period following my second dose	1558	97.0
Yes, but I haven't yet had a period following my second do	48	3.0
**Which vaccine did you get for your second dose**		
AstraZeneca	250	15.6
Pfizer	1326	82.6
Moderna	30	1.9
**On which day of your menstrual cycle did you get your second dose (where day 1 is the first day of your period)**		
1–7	340	21.2
8–14	328	20.4
15–21	409	25.5
After 21	529	32.9
**Q1. After receiving the second dose, your next period was**		
Lighter than usual	334	20.8
Heavier than usual	275	17.1
The same as usual	997	62.1
**Q2. After receiving the second dose, your next period was**		
Earlier than usual	287	17.9
On-time	923	57.5
Later than usual	396	24.7
**Q3. After receiving the second dose, the cramps you experienced in your next period was**		
Worse than usual	430	26.8
Less bad than usual	143	8.9
The same as usual	1033	64.3
**Q4. After receiving the second dose, premenstrual syndrome) PMS you experienced in your next period was**		
Worse than usual	386	24.0
Less bad than usual	110	6.8
The same as usual	1110	69.1
**Have you ever noticed a change in your periods after any other vaccine**		
No	1565	97.4
Yes, after the flu vaccine	9	0.6
Yes, after the human papillomavirus (HPV) vaccine	2	0.1
Yes, after another vaccine	30	1.9

There were no significant associations found between the type of COVID-19 vaccine and the impact on the menstrual cycle, either for the first dose or for the second dose (*P*-values > 0.05) (Table 4).

**Table 4:** The association between the type of COVID-19 vaccine and the impact on the menstrual cycle

Variable	Vaccine type							

	First dose				Second dose			
	
	AstraZeneca	Pfizer	Moderna	P-value	AstraZeneca	Pfizer	Moderna	P-value
**Q1. After receiving the COVID-19 vaccine, your next period was**								
				0.12				0.09
Lighter than usual	149	423	2		47	284	3	
Heavier than usual	86	246	6		50	223	2	
The same as usual	392	1025	9		153	819	25	
**Q2. After receiving the COVID-19 vaccine, your next period was**								
				0.26				0.77
Earlier than usual	98	294	1		43	241	3	
On-time	368	919	10		147	756	20	
Later than usual	161	481	6		60	329	7	
**Q3. After receiving the COVID-19 vaccine, the cramps you experienced in your next period was**								
				0.32				0.61
Worse than usual	158	456	5		73	350	7	
Less bad than usual	41	141	0		24	118	1	
The same as usual	428	1097	12		153	858	22	
**Q4. After receiving the COVID-19 vaccine, premenstrual syndrome) PMS you experienced in your next period** **was**								
				0.06				0.89
Worse than usual	138	409	6		61	319	6	
Less bad than usual	26	113	0		18	91	1	
The same as usual	463	1172	11		171	916	23	

**Figure 1:**
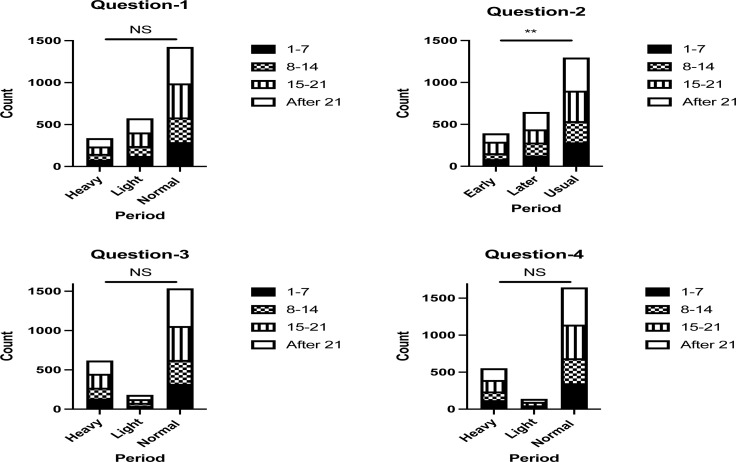
The association between first dose vaccination day and the impact on the menstrual cycle

A significant association was found only between the first dose vaccination day and the impact on the menstrual cycle in the second question of “After receiving the COVID-19 vaccine, your next period was” (*P*-value ≤ 0.05). In contrast, no significant associations were found between the first dose vaccination day and the impact on the menstrual cycle in the other questions (*P*-values > 0.05) (Figure 1).

**Figure 2:**
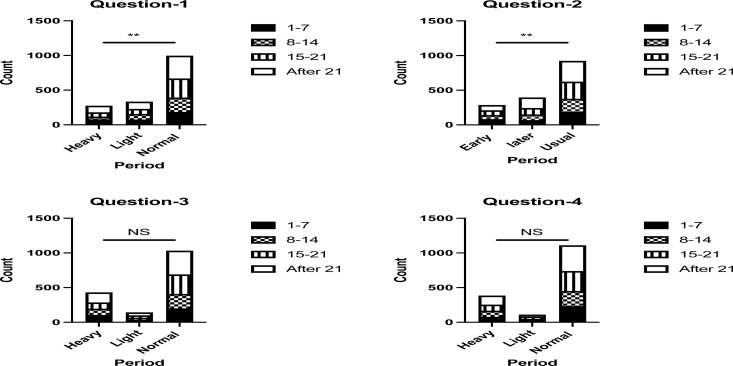
The association between the second dose vaccination day and the impact on the menstrual cycle

Significant associations were found between the second dose vaccination day and the impact on the menstrual cycle in the first and second questions of “After receiving the COVID-19 vaccine, your next period was”, and “After receiving the second dose, your next period was,” respectively (*P*-values ≤ 0.05). In contrast, no significant associations were found between the second dose vaccination day and the impact on the menstrual cycle in the third and fourth questions (*P*-values > 0.05) (Figure 2).

## Discussion

To the best of our knowledge, the current study is the first to investigate the effect of the COVID-19 vaccine on the menstrual cycle among females in Saudi Arabia. Our study showed that more than half of the study participants had not noticed any menstrual abnormalities after being vaccinated against COVID-19, after both the first and second doses. In addition, the COVID-19 vaccine had no impact on the menstrual cycle among the participating females, either for the first or second doses. However, a percentage of females reported that after receiving the first dose, their next period was heavier than usual (14.5%), later than usual (27.7%), the cramps were worse than usual (26.5%), and the premenstrual syndrome was worse than usual (23.7%). As well, the percentage of females who reported that their next period was heavier than expected after receiving a second dose was (17.1%), later than usual was (24.7%), and cramps were worse than usual (26.8%). The premenstrual syndrome was worse than usual among 24.0% of women, indicating that menstrual problems following vaccination significantly impacted the quality of life of the participating females.

A recent study conducted in the MENA Region by Nadia Muhaidat and her colloquies showed that menstrual symptoms were recorded by 66.3% of women following COVID-19 vaccination, with 46.7% experiencing the symptoms after the first dosage (11).

The pattern of menstrual bleeding is a vital sign of reproductive health (12). On the other hand, menstrual symptoms such as perimenstrual mood disorders, menstrual cramps, and excessive menstrual flow are frequent gynecological issues. In a nationwide survey involving 42,879 healthy premenopausal Dutch women (13), 53.7% complained of heavy bleeding, 77.3% complained of perimenstrual psychological complaints, and 85.4% complained of monthly pains nationwide, 42,879 healthy premenopausal Dutch women (14). This is due to the female menstrual cycle being influenced by a variety of causes that can be temporary, such as infections, weight gain, anxiety, hormonal changes, and periods of psychological stress, or long-term, such as endocrinopathies and polycystic ovarian syndrome, which require treatment (14–16).

Stressors can activate the hypothalamic-pituitary-gonadal axis, causing hormone release to become irregular. These menstrual changes can negatively influence a woman's quality of life, limiting her ability to work and attend school, preventing her from achieving her goals, and interfering with her social and professional activities, all of which can add to her stress (17,18). The COVID-19 pandemic was one form of stress that took the world by storm, with multiple studies showing an increase in menstrual cycle anomalies during the pandemic compared to before (14,15,19,20).

In the literature, the occurrence of irregular menstruation varies from 5% to 35.6%, depending on occupation, age, and geographic location (21–26). The percentage of females who had irregular menstrual cycles before vaccination in our study was within that range, with 5.6% having irregular menstrual periods. However, after immunization, a considerable number of women suffered irregular periods. Even after controlling for variations in menstrual bleeding during the COVID-19 pandemic, only the first dose vaccination day and the influence on the menstrual cycle were found to have a sincere relationship in the second question, “What was your next period after receiving the COVID-19 vaccine?” Furthermore, significant associations were found between the second dose vaccination day and the impact on the menstrual cycle in the first and second questions of “After receiving the COVID-19 vaccine, your next period was,” and “After receiving the first dose, your next period was.” Our findings align with a recently published study of 39,129 individuals in the United States, which found that 42% experienced increased bleeding following immunization (27). Also, according to the survey by Nadia Muhaidat et al., nearly a third of the individuals, 35.3%, had menstrual alterations during the COVID-19 pandemic before vaccination. However, 66.3% of women had irregular periods due to the vaccination (11). According to another study, only 20% of 4989 premenopausal vaccinated women in the U.K. did not report any menstrual cycle anomalies four months following their initial COVID-19 vaccination injection (5).

Our study's key strength is that, despite numerous reports on social media platforms and news channels, it is one of the first to address the topic of post-vaccination menstrual irregularities in Saudi Arabia (21,28,29). In our study, we recruited a sufficient sample size of vaccinated Saudi Arabian women, allowing us to assess the prevalence of post-vaccination menstrual irregularities in a conservative society, which is a delicate topic. It also serves as a baseline for evaluating the influence of COVID-19 vaccinations on women's menstrual cycles. Another strength is the data gathering tool, which has been carefully built and validated to capture the situation accurately and thoroughly. As a result, we believe our findings adequately reflect the menstrual irregularities that women experience after vaccination.

Nevertheless, there are some flaws in the research. The study's cross-sectional methodology hindered our capacity to discern causal correlations. Furthermore, because persons with menstruation issues may be more interested in participating in the study, self-reported data extraction has a higher risk of recall bias or self-selection. Furthermore, an internet-based survey may have under- or over-represented certain target groups, particularly elderly individuals with low internet access or technological expertise. In addition, mostly younger females with internet access would participate in this study; therefore, sampling bias is the main restriction in this study. Nonetheless, given the current epidemiological situation, online questionnaires are the most effective and secure data collection tool. Furthermore, due to the quick addition of publications to the COVID-19 literature, there are constraints to including the most recent papers.

In conclusion, the current study presents preliminary evidence that females who have received COVID-19 vaccines may have menstrual cycle irregularities such as prolonged menstrual periods. Such abnormalities may impact a female's daily activities, lowering their overall quality of life. Our study also suggests that these symptoms may be self-limiting and temporary. Nonetheless, the COVID-19 pandemic's shifted priorities make females less likely to engage in health-seeking behaviors. As a result, it is critical to notify healthcare providers and women about menstrual irregularities following vaccination. No significant associations were found between the type of COVID-19 vaccine and the impact on the menstrual cycle, either for the first or second dose. Further research is needed concerning how COVID-19 vaccination causes menstrual abnormalities is required.
